# A potential biomarker for treatment stratification in psychosis: evaluation of an [^18^F] FDOPA PET imaging approach

**DOI:** 10.1038/s41386-020-00866-7

**Published:** 2020-09-22

**Authors:** Mattia Veronese, Barbara Santangelo, Sameer Jauhar, Enrico D’Ambrosio, Arsime Demjaha, Hugh Salimbeni, Jin Huajie, Paul McCrone, Federico Turkheimer, Oliver Howes

**Affiliations:** 1 Department of Neuroimaging, Institute of Psychiatry, Psychology & Neuroscience, King’s College London, London, UK; 2 Department of Psychosis Studies, Institute of Psychiatry, Psychology & Neuroscience, King’s College London, London, UK; 3 Psychiatric Neuroscience Group, Department of Basic Medical Sciences, Neuroscience and Sense Organs, University of Bari “Aldo Moro”, Bari, Italy; 4 Faculty of Engineering, Department of Computing, Imperial College London, South Kensington Campus, London, UK; 5 King’s Health Economics, Health Service and Population Research Department, Institute of Psychiatry, Psychology & Neuroscience, King’s College London, London, UK; 6 Faculty of Education, Health and Human Sciences, University of Greenwich, London, UK; 7 MRC London Institute of Medical Sciences, Hammersmith Hospital, London, W12 0NN UK; 8 Institute of Clinical Sciences (ICS), Faculty of Medicine, Imperial College London, Du Cane Road, London, W12 0NN UK

**Keywords:** Predictive markers, Biomarkers

## Abstract

[^18^F]FDOPA PET imaging has shown dopaminergic function indexed as *K*_i_^cer^ differs between antipsychotic treatment responders and non-responders. However, the theragnostic potential of this biomarker to identify non-responders has yet to be evaluated. In view of this, we aimed to evaluate this as a theragnostic test using linear and non-linear machine-learning (i.e., Bernoulli, support vector, random forest and Gaussian processes) analyses and to develop and evaluate a simplified approach, standardised uptake value ratio (SUVRc). Both [^18^F]FDOPA PET approaches had good test-rest reproducibility across striatal regions (*K*_i_^cer^ ICC: 0.68–0.94, SUVRc ICC: 0.76–0.91). Both our linear and non-linear classification models showed good predictive power to distinguish responders from non-responders (receiver operating curve area under the curve for region-of-interest approach: *K*_i_^cer^ = 0.80, SUVRc = 0.79; for voxel-wise approach using a linear support vector machine: 0.88) and similar sensitivity for identifying treatment non-responders with 100% specificity (*K*_i_^cer^: ~50%, SUVRc: 40–60%). Although the findings were replicated in two independent datasets, given the total sample size (*n* = 84) and single setting, they warrant testing in other samples and settings. Preliminary economic analysis of [^18^F]FDOPA PET to fast-track treatment-resistant patients with schizophrenia to clozapine indicated a potential healthcare cost saving of ~£3400 (equivalent to $4232 USD) per patient. These findings indicate [^18^F]FDOPA PET dopamine imaging has potential as biomarker to guide treatment choice.

## Introduction

Schizophrenia and related psychotic disorders are common mental disorders and amongst the leading causes of global disability [1–3]. Antipsychotic drugs are central to their treatment [4, 5]. However, about one-third of patients show limited response to first-line antipsychotic treatment, often from illness onset [6, 7]. Poor response is associated with increased health burden, higher costs and longer hospital stays [8]. Clozapine is an alternative treatment that is effective in people resistant to first-line antipsychotic drugs [9]. Moreover, clozapine treatment is associated with reduced mortality, healthcare costs and functional outcomes [10–14]. However, clinical guidelines around the world recommend its use is restricted to non-responders because of the risk of side effects and the need for blood monitoring [15]. As there is currently no way to differentiate non-responders from responders, clinical guidelines recommend a series of empirical treatment trials to determine if a patient is a non-responder, before then initiating clozapine. In practice this leads to long delays, on average over 4 years, before initiation of clozapine [16]. There is thus a clinical need for a biomarker to identify non-responders early to guide treatment choice.

Both the role of dopamine hyperactivity in the pathoetiology of psychosis [17–21], and findings that antipsychotic drugs act by blocking dopamine [4, 22] indicate that molecular imaging of the striatal dopamine innervation is a candidate biomarker for predicting treatment response. A number of studies have shown that striatal dopamine synthesis capacity, as measured by [^18^F]FDOPA PET imaging, is elevated in schizophrenia [23], schizophreniform psychoses [24] and people at clinical high risk for psychosis (UHR) [24–26], and linked to subsequent development of psychosis [24–26]. Moreover, dopamine synthesis capacity has been found to distinguish patients who have responded to standard antipsychotic drugs from non-responders [27] and both this and synaptic dopamine levels have been shown to predict response to antipsychotic treatment [20, 28, 29]. This evidence suggests that [^18^F]FDOPA PET imaging of the striatal dopamine innervation could be used as a neurochemical basis to stratify patients into those likely to respond and those unlikely to respond to first-line antipsychotic drugs. Timely stratification would enhance patient welfare by enabling more rapid treatment responses, while bringing a considerable economy to the healthcare system.

However, the theragnostic potential of this method to identify patients who will respond to first-line treatment has yet to be evaluated. In view of this, our first aim was to determine the sensitivity and specificity of [^18^F]FDOPA PET imaging to distinguish treatment responders from non-responders. For a diagnostic test to be clinically useful it also needs to be practical. One potential limitation of the [^18^F]FDOPA PET imaging used in prior studies is the long duration of the scans, about 95 min. To address this, we aimed to evaluate a shorter, simplified protocol [^18^F]FDOPA PET imaging protocol and compare its accuracy with the full 95-min scans. Finally, we conducted a preliminary cost analysis using [^18^F]FDOPA PET to identify non-responders to first-line antipsychotic treatment.

## Methods and materials

The most common approach to [^18^F]FDOPA PET imaging uses continuous dynamic acquisition, with the scanning beginning with the tracer injection and lasting for 90–95 min, during which the participant is required to lie still in the PET scanner. A 60-min acquisition had also been considered; however, a correlation analysis indicated that the 95-min acquisition is more reliable (Supplementary Tables 1 and 2).

Compared to this dynamic [^18^F]FDOPA PET acquisition, the simplified protocol consists of a brief single-frame acquisition (10–15 min) and a simplified index of FDOPA uptake (standardised uptake value ratio (SUVRc)), defined as the ratio of the tracer activity in the striatum to that of the cerebellum, as a proxy for dopamine synthesis capacity using the standard dynamic approach. This approach is similar to simplified [^18^F]FDOPA PET scanning methods that have already been used to distinguish patients with early Parkinson disease (PD) from healthy volunteers [30, 31].

To explore the generalisability of the simplified [^18^F]FDOPA PET imaging beyond the specifics of the datasets included in this study, we also tested its sensitivity to important experimental variables (the tracer injected dose and specific activity (SA) as well as the length of PET scan acquisition).

### Datasets

The data presented are a new analysis of two different [^18^F]FDOPA PET imaging datasets that have been previously published [28, 32]. *Dataset1* [28] consists of 26 first-episode psychosis patients, who were scanned prior to antipsychotic treatment and three minimally treated for <2 weeks, and 14 age matched healthy volunteers to enable normative comparisons. Following the scans, all patients began treatment with a first-line antipsychotic medication, selected upon consultation with their psychiatrist and without reference to PET results. Treatment choice was made by the patient in consultation with their psychiatrist [28]. Treatment was titrated to a therapeutic dose (based on the Maudsley Prescribing Guidelines) and patients all received follow-up to at least 6 months to determine response status. To be included patients were required to show good adherence to treatment defined as taking more than 80% of prescribed antipsychotic doses in line with recommendations and other studies [15, 33, 34]. To assess concordance with antipsychotic medication, a multisource approach was used. This required evidence of adequate adherence on at least two of the following: antipsychotic plasma levels, pharmacy and electronic medical dispensing records and report from the patient and an independent source (family member/caregiver or healthcare professional). Adequate concordance was defined as taking a minimum of 80% of prescribed doses, in line with consensus recommendations [34].

Treatment response was defined as >50% reduction in PANSS total symptom severity rating from baseline to follow-up and response sustained over at least 6 months, in line with recommendations for response in early course of the illness [5, 15]. Treatment non-response was defined as <50% reduction in PANSS total symptom severity rating from baseline and no evidence of response over at least 6 months. Of the 26 patients, 13 met criteria for treatment response and 13 met criteria for non-response [35, 36].

*Dataset2* [32] consists of 12 treatment non-responsive patients with schizophrenia, 12 treatment-responsive patients with schizophrenia and 12 age matched healthy volunteers for normative comparisons. Treatment non-response was defined as meeting modified Kane criteria for treatment resistance in schizophrenia [37]. Treatment response was defined as meeting the Remission in Schizophrenia Working Group criteria for treatment remission [38]. Full details of inclusion criteria, medication status of the patients and clinical assessment are reported in the original references [28, 32]. For *Dataset1* and *Dataset2*, both treatment responders and non-responder patients show no significant difference for age, gender, weight, ethnicity and cigarette smoking with the corresponding control groups.

An additional test-retest dataset (*Dataset3*), consisting of eight healthy controls (mean age 23.6 ± 3.5 years, 5 male, injected dose of ~150 MBq) scanned twice, ~2 years apart, was also used for the reliability analysis of the proposed simplified [^18^F]FDOPA PET imaging protocol. Full details on the research protocol and subject inclusion criteria are reported in the original reference [39].

A summary of subject demographics for all the three datasets is reported in Supplementary Table 3. All studies were approved by the local research ethics committee. After full description of the respective studies, all participants gave written informed consent to participate, and consent for data to be used in further analyses.

### PET imaging

The experimental protocol for the three datasets used the same standard approach [34, 40–42], although the target injected radioactivity was ~150 MBq for *Dataset1* and *Dataset3*, and ~180 MBq for *Dataset2*. All participants received carbidopa (150 mg) and entacapone (400 mg) orally 1 h before imaging. Both drugs are used to increase the signal-to-noise ratio (SNR) of the tracer uptake in brain tissue by reducing the peripheral formation of radiolabelled dopamine and 3-O-methyl-[^18^F]fluorodopa, the brain-penetrating metabolite, respectively [43–45].

The [^18^F]FDOPA tracer was administered by intravenous bolus injection after acquisition of a brain CT scan for attenuation correction. For *Dataset1* and *Dataset2*, [^18^F] FDOPA PET imaging was performed dynamically in 3-dimensional mode. Data were acquired using a Siemens Biograph 6 HiRez PET scanner (Siemens, Erlangen, Germany) for *Dataset1*, a Siemens/CTI ECAT HR + 962 PET scanner (Siemens, Erlangen, Germany) for *Dataset2* and a Siemens/CTI ECAT/EXACT3D (Knoxville, Tennessee) for *Dataset3*. The three machines have similar spatial resolution (4.5 ± 0.24, 4.8 ± 0.2 and 4.5 ± 0.2 mm respectively) and comparable sensitivity (4.2, 4.2 and 4.5 cps/kBq). In *Dataset1* PET data were binned in 32 frames of increasing duration over the 95 min scans (frame intervals in seconds: 8 × 15, 3 × 60, 5 × 120, 16 × 300). Emission data for *Dataset2* were obtained as 26 frames of increasing duration over 90 min (frame intervals in seconds: 1 × 30, 4 × 60, 3 × 120, 3 × 180, 15 × 300). In *Dataset3* PET data were acquired in list mode for 95 min, re-binned into 26 frames (frame intervals in seconds: 1 × 30, 4 × 60, 3 × 120, 3 × 180, 15 × 300).

### Image analysis

[^18^F]FDOPA PET imaging analyses used the approach reported in previous papers [34, 40–42]. For all datasets, motion correction was performed first realigning frame-to-frame nonattenuated dynamic images to a single reference frame. The transformation parameters were then applied to the corresponding attenuated-corrected frames. The realigned frames were finally summed to create motion-corrected dynamic images. A tracer-specific template [32, 41] and atlas defining the striatum and cerebellum (see [46]) were co-registered onto each subject’s PET image using a combination of Statistical Parametric Mapping 8 (https://www.fil.ion.ucl.ac.uk/spm/) and in-house Matlab-based scripts. The striatal atlas included limbic, associative and sensorimotor subdivisions based on the predominant origin of projections to sub-regions of the striatum and given evidence that dopaminergic alterations are most marked in the associative striatum in schizophrenia [47]. The main outcome parameter was *K*_i_^cer^ (min^−1^), calculated using the Patlak–Gjedde graphical approach with the cerebellum as reference region, as this region shows negligible dopamine synthesis [48]. *K*_i_^cer^ parametric images of the brain were constructed from motion-corrected images using a wavelet-based approach [49]. The parametric image for each participant was then normalised into Montreal Neurological Institute standard space using the participant’s PET summation image and the [^18^F]FDOPA PET template. This analysis pipeline is completely automated and operator independent, leading to reproducible (ICC > 0.8 [39]) and replicable results [27, 28, 50].

The simplified index of FDOPA uptake (SUVRc) was then defined by the ratio of the tracer activity in the striatum and its functional subdivisions to that in the reference region, using the same atlas as used for the dynamic scan. Consistent with the dynamic analysis, the cerebellum was used as the reference region. To test the sensitivity of this metric to time point after tracer injection, SUVRc was generated for the striatum and each functional striatal subdivision for a 10-min time frame at 60, 75 and 90 min after injection. These timepoints were chosen because the Gjedde–Patlak plot is linear within this time window, and it is the linear portion of the Gjedde–Patlak plot that is used to derive *K*_i_^cer^ [48].

Classification analysis was limited to the whole striatum and its associative subdivision. Previous studies have identified the associative striatum as the striatal region with the most marked dopaminergic alteration in psychosis [51]. The associative striatum was also reported to be the main locus for differentiating responders from non-responders [32].

We investigated the impact of reducing or increasing the length of the simplified PET scan acquisition by 5 min using *Dataset3*. The functional striatal atlas was co-registered with the pseudo-static PET images obtained from taking the mean signal of the original dynamic scans over 5, 10 and 15 min, respectively starting at 75 min from the tracer injection. The co-registration was performed using Statistical Parametric Mapping 8 (https://www.fil.ion.ucl.ac.uk/spm/). The SNR was defined by the ratio of the mean signal in the striatum and the standard deviation of the brain signal outside the striatal regions [52].

### Preliminary health economic analysis

We investigated the economic sustainability of the biomarker if it was offered as screening test in a representative population of 1000 individual with first-episode psychosis.

We used the following assumptions in the calculation:

1. Cost per scan £3000 ($3900) (https://www.xe.com/currencyconverter/), for a static 10–15 min FDOPA scan in a clinical nuclear medicine setting inclusive of tracer delivery. This is based on the cost of commercial delivery of tracer plus scanning time and all other imaging centre costs as provided by current providers, although for national health system it can be cheaper [53].
2. In total, 33% of patients do not respond to conventional antipsychotics [54].
3. Potential annual healthcare savings of ~ £24,000/year based on the healthcare costs of a patient with active psychosis of £39,141/year and the healthcare costs of a patient with no active psychotic symptoms of £15,086 [55–58].
4. Cost of treating patients with clozapine (costs of medication, the monitoring service and management of neutropenia occurring in 3% of patients) for all the patients classified by the biomarker as non-responders [59, 60].
5. A mean delay to clozapine of 4 years in current clinical practice [61].
6. In total, 50% of patients with treatment-resistant schizophrenia achieve clinical response with clozapine treatment [62].

Based on these assumptions we calculated the potential annual savings of identifying a patient who is a non-responder to first-line antipsychotic treatment and starting them on clozapine within a month from the diagnosis and the minimal statistical performance of the biomarker to be cost effective in clinical use.

### Statistical analysis

Statistical analyses were performed using Prism, version 7 (https://www.graphpad.com/scientific-software/prism/), and SPSS, version 24 (https://www.ibm.com/analytics/spss-statistics-software). Normality of distribution was assessed using Shapiro–Wilkes test. Test-retest reliability of SUVRc was calculated using the intraclass correlation coefficient (ICC) [63]. This model estimates the correlation between individual [^18^F]FDOPA SUVRc values between scan sessions using a two-way ANOVA with random subject effects and fixed session effects. This model was chosen over a one-way random model because SUVRc values were ordered into two sessions (test and retest scan), and the two-way random model additionally accounts for systematic sources of variance associated with session effects [64]. Test-retest reproducibility was calculated as the percentage test-retest difference (VAR absolute value):

$${\mathrm{VAR}} = 2 \times \left| {\mathrm{Retest} - \mathrm{Test}} \right|/(\mathrm{Test} + \mathrm{Retest}) \times 100.$$

The agreement between SUVRc values and *K*_i_^cer^ values determined using a full scan was studied with a correlation analysis. In this analysis, *K*_i_^cer^ and SUVRc of all participants of *Dataset1* and *Dataset2* were correlated within each functional striatal ROI by computing Pearson’s product moment correlations for normally distributed data and Spearman’s rank correlation coefficients for non-normally distributed data. Secondly, we computed the responder vs. non-responder Cohen’s d effect size to identify the degree of group difference in dopamine synthesis capacity as returned by SUVRc in comparison to *K*_i_^cer^.

To investigate the statistical power of SUVRc to identify non-responder patients from the entire patient pool, an analysis of receiver operating characteristics (ROC) curves was performed. *Dataset1* and *Dataset2* were analysed separately and the SUVRc metrics computed at different timepoints were compared with *K*_i_^cer^ performances. The ROC area under the curve (AUC) was used as the performance index. As an additional performance metric, we extracted the biomarker sensitivity when the classification threshold was set at 100% specificity, corresponding to the fraction of non-responders correctly identified without any mislabelling of the responder group as non-responders. The classification threshold was set at this high level of specificity to avoid treatment with clozapine in someone who would respond to an alternative antipsychotic, given the monitoring and potential side effect burden associated with clozapine [65].

### Machine-learning approaches applied to voxel-level data

The primary analyses of the discriminative power used *K*_i_^cer^ values from the atlas-based region-of-interests for the whole striatum and its functional subdivisions, which represents an average value of all voxels within the region-of-interest. However, it is possible that other characteristics of the data, such as *K*_i_^cer^ values in individual voxels and/or non-linear relationships within the data, are important in discriminating between responders and non-responders; i.e., there might be sub-anatomical areas in the striatum that have better predictive power for the treatment response.

In view of this, we also investigated the potential of using machine-learning models to predict the non-responder status using the voxel-based PET data across the striatum to improve the discriminatory power compared to using mean striatal values from the region-of-interest analyses. We used the 2847 voxels from the striatum segmentation mask, with no additional pre-processing, and applied a leave-one-out cross validation approach to train and evaluate the classifiers.

We evaluated a variety of linear and non-linear models, using leave-one-out cross validation to estimate the generalisation of performance. The predictor, *x*_i_, is the voxel-level dopamine synthesis values (flattened to a vector representation) and the target, *y*_i_, is the responder status, with 1 for non-responder and −1 for responders. We considered two linear models with loss functions of the form:

$$\mathop {\sum}\limits_{i = 1}^N l (y_i,f_i) - \alpha \left\| W \right\|^2\;\;\;\;{\mathrm{with}}\;\;\;\;f_{i}=\; Wx_{i}+b,$$

where *N* is the number of the subjects, *α* is a regularisation parameter (fixed to 1, as good trade-off between data fitting and regularisation [66]), *W* is a vector of weights to be learned and *b* is a bias parameter to be learned.

The first was a Bernoulli observations model with a logistic link function [67]. The second was a linear Support Vector Machine [68], which uses a high loss to maximise the margin between classes. We also evaluated two non-linear models. First, we used a Random Forests approach [69], which avoids overfitting by averaging base classifiers in an ensemble technique known as bootstrap aggregating. The base classifiers are decision trees, which are de-correlated through data subsampling and feature subsampling. Second, we evaluated a Gaussian processes approaches [66], which uses kernelized Bayesian linear models, with the Squared Exponential kernel and Bernoulli loss with probit link function, fit using variational inference. All models were implemented in sklearn [70], except the Gaussian process, which was implemented in gpflow [71].

### Supplementary analyses: sensitivity to experimental variables

To explore the generalisability of SUVRc beyond the specifics of the datasets included in this study, we tested its sensitivity to the tracer injected dose and SA by using Spearman’s correlation analysis.

## Results

### Static vs. dynamic FDOPA

**Fig. 1:**
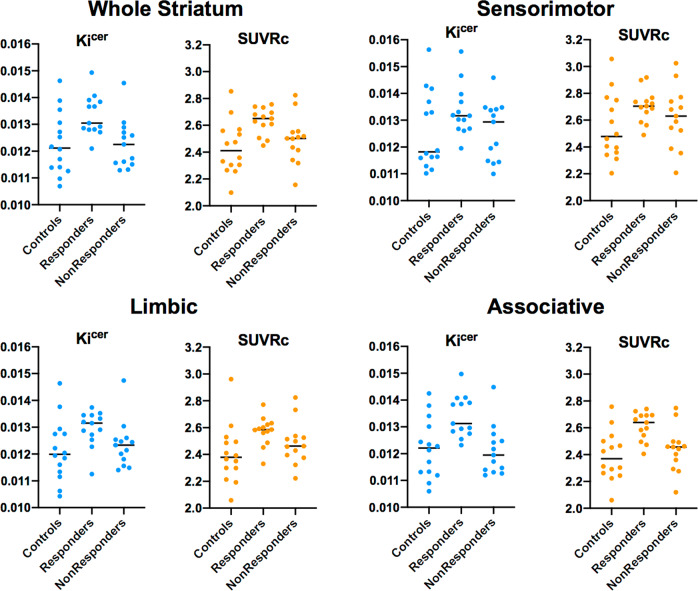
Gold standard index of dopamine synthesis capacity (*K*_i_^cer^ using 95 min acquisition) and the simplified index of FDOPA uptake (SUVRc with a 15 min static acquisition 75 min after tracer injection) in striatum and its subdivisions. These values are from each group (controls, responders and non-responders) of Dataset1.

**Fig. 2:**
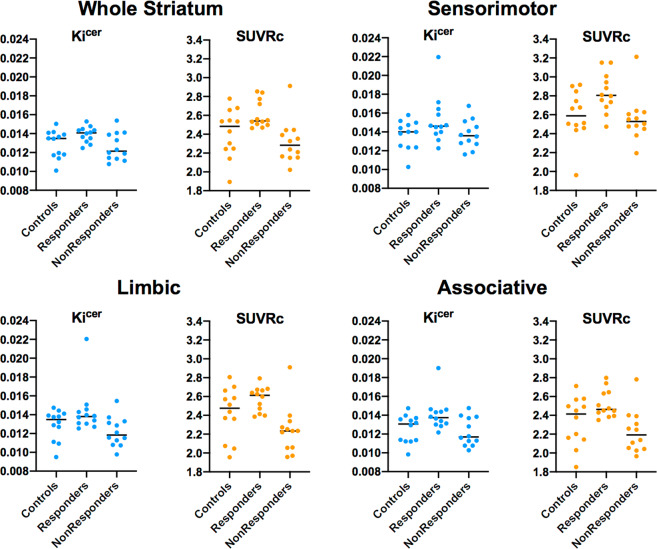
Gold standard index of dopamine synthesis capacity (*K*_i_^cer^ using 95 min acquisition) and the simplified index of FDOPA uptake (SUVRc with a 15 min static acquisition 75 min after tracer injection) in striatum and its subdivisions. These values are from each group (controls, responders and non-responders) of Dataset2. For consistency, dataset 2 was reprocessed with the same analysis pipeline used for dataset 1, which may explain differences between data points shown here and the original publication [32].

For both datasets, the standard dynamic measure of dopamine synthesis capacity (*K*_i_^cer^) was significantly correlated with the simplified index of FDOPA uptake (SUVRc) in whole striatum (Spearman’s rho from 0.60 to 0.89, all *p* values < 0.001). The correlation between striatal SUVRc and *K*_i_^cer^ at 75 min after injection was the highest (Spearman’s rho from 0.79 to 0.89), while it was lowest for SUVRc calculated at 90 min (Spearman’s rho from 0.75 to 0.85). Significant correlations were also observed when considering only the patient groups (both responders and non-responders) in both datasets, with Spearman’s rho ranging from 0.74 to 0.87 at 60 min, from 0.74 to 0.82 at 75 min and from 0.60 to 0.83 at 90 min after tracer injection. Full details of the correlation analysis results are reported in Supplementary Tables 4 and 5 and Supplementary Fig. 1. Mean values of *K*_i_^cer^ and SUVRc of *Dataset1* and *Dataset2* are reported in Figs. 1 and 2.

For *Dataset1* (in which one of the three minimally treated patients was a responder) we observed significant differences in SUVRc between responders and non-responders with small to very large Cohen’s d effect sizes (mean ± SD: 0.77 ± 0.37), with the maximum obtained for the associative striatal subdivision at 75 min from tracer injection (Cohen’s d: 1.32) and the minimum obtained for the sensorimotor striatal subdivision at 90 min from tracer injection (Cohen’s d: 0.17). For *Dataset2*, we also observed significant differences in SUVRc between responders and non-responders, with moderate to very large Cohen’s d effect sizes (mean ± SD: 0.69 ± 0.08). As for *Dataset1*, the highest values were obtained for the striatum and the associative striatal subdivision at 75 min from tracer injection (both with Cohen’s d effect sizes of 0.79), while the smallest difference was obtained for the sensorimotor striatal subdivision at 60 min from tracer injection (Cohen’s d: 0.57). Overall the whole striatum and its associative striatal subdivision were the areas where the effect sizes for the differences between responders and non-responders were the highest (Cohen’s d values in the striatum: from 0.62 to 1.09; Cohen’s d values in the associative striatum: from 0.64 to 1.32).

Scan timing did have an impact on effect size for SUVRc. The lowest values were obtained at 90 min from tracer injection (mean ± SD: 0.55 ± 0.22) while the highest ones were obtained at 75 min from tracer injection (mean ± SD: 0.81 ± 0.24) in both datasets. Full details of the Cohen’s d effect size are reported in Supplementary Table 6.

### Test-retest analysis

Mean percent test-retest differences and reliability for SUVRc values in the striatum and in the functional striatal subdivisions are presented in Supplementary Table 7. Overall, the reproducibility of SUVRc was excellent (%VAR < 4), regardless of the time after injection and the striatal area in which it was measured. We also observed good test-retest reliability values in all the functional striatal subdivisions (ICC: 0.76–0.91).

### Sensitivity to experimental variables

There was no correlation between SUVRc and either injected dose (*p* = 0.89) or SA (*p* = 0.97) (Supplementary Fig. 2). In contrast, there was a significant effect of scan duration on SUVRc maps (Supplementary Fig. 3): the signal-to–noise ratio increased as the acquisition length was extended (mean ± SD: for 5 min acquisition 0.76 ± 0.0.48; for 10 min acquisition 0.87 ± 0.49; for 15 min acquisition 0.88 ± 0.55).

### Classification of treatment response: region-of-interest analyses

**Fig. 3:**
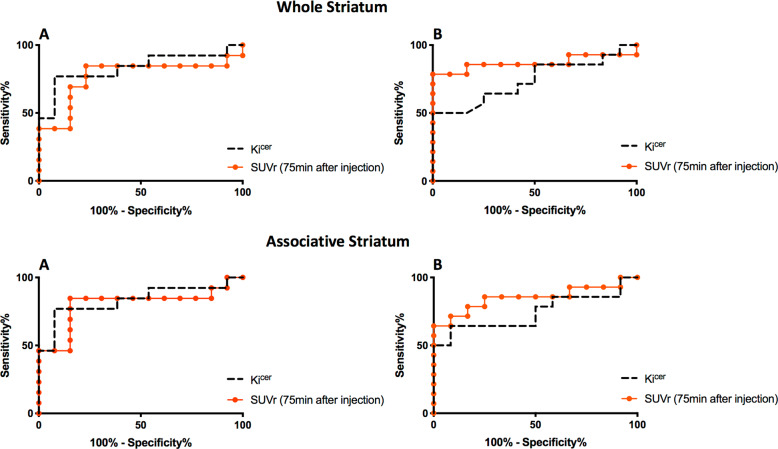
Receiver Operating Characteristic (ROC) curves for the classification of patients into responders and non-responder groups using [^18^F]FDOPA PET imaging analysed using the simplified index of FDOPA uptake (SUVRc) or the standard dynamic measure of dopamine synthesis capacity (*K*_i_^cer^) in the whole striatum and associative striatal subdivision. This analysis shows the results from Dataset1 (**a**) and Dataset2 (**b**).

The ROC curves for SUVRc from 75 min after tracer injection and *K*_i_^cer^ values in the whole striatum and in its associative striatal subdivision are shown in Fig. 3, while the ROC curves for SUVRc from 60 and 90 min after tracer injection are reported in Supplementary Fig. 4. AUC estimates and the sensitivity at 100% specificity are reported in Table 1.

**Table 1:** Receiver Operating Characteristic (ROC) curves and sensitivity analyses for the classification of patients into antipsychotic responders and non-responders using the simplified index of FDOPA uptake (SUVRc) and the gold standard (*K*_i_^cer^) index of dopamine synthesis capacity in the striatum and associative striatal subdivision for Dataset1 and Dataset2.

	AUC ROC				Sensitivity at 100% specificity cut-off			
	Whole striatum		Associative		Whole striatum		Associative	
	Dataset1	Dataset2	Dataset1	Dataset2	Dataset1 (%)	Dataset2 (%)	Dataset1 (%)	Dataset2 (%)
SUVRc 60 min	0.70*	0.86*	0.74*	0.83*	31	79	31	64
SUVRc 75 min	0.77*	0.87*	0.80*	0.85*	39	79	46	64
SUVRc 90 min	0.66^NS^	0.81*	0.74*	0.81*	23	43	23	36
*K*_i_^cer^	0.83*	0.74*	0.87*	0.75*	46	50	61	50

The *p* value tests the null hypothesis that the area under the curve really equals 0.50 (chance).

*AUC* area under the curve.

^*^p < 0.05; NS *p* > 0.05.

For *Dataset1* we observed ROC AUC estimates varying from 0.66 to 0.80 (mean ± SD: 0.74 ± 0.05). For *Dataset2* the AUC estimates varied from 0.74 to 0.87 (mean ± SD: 0.84 ± 0.03). The best estimates were observed at 75 min after tracer injection (mean ± SD: 0.82 ± 0.04) for both the striatum and the associative striatal subdivision.

For *Dataset1* the *K*_i_^cer^ sensitivity to identify non-responders at 100% specificity varied from 46 to 61% (mean ± SD: 53.5 ± 10.6), whereas the SUVRc sensitivity varied from 23 to 46% (mean ± SD: 32.2 ± 9.0). For *Dataset2* we observed *K*_i_^cer^ sensitivity at 100% specificity of 50% and SUVRc sensitivity varying from 36 to 79% (mean ± SD: 60.8 ± 17.9).

### Classification of treatment response: machine-learning voxel-based analyses

We also investigated the potential of using machine-learning approaches applied to striatal voxel-wise data to distinguish responders from non-responders. For both the linear and non-linear methods the AUC estimates are given in Table 2. A consistent pattern was a higher score in *Dataset2* than *Dataset1*. For *Dataset1* none of the models surpassed the ROI classifier as measured by ROC AUC (mean ± SD AUC for linear SVM = 0.71 ± 0.001, for SVM with radial basis function = 0.45 ± 0.025, for Random Forest = 0.70 ± 0.008, Linear = 0.74, for Kernel K-nearest with 2 neighbours Knn(2) = 0.63, for Kernel K-nearest with 3 neighbours Knn(3) = 0.68, and for Gaussian process approach = 0.64). For *Dataset2* the linear model and linear Support Vector Machine model provided superior classification than the ROI classifier as measured by ROC AUC (mean ± SD AUC for linear SVM = 0.88 ± 0.001, for SVM with radial basis function = 0.80 ± 0.007, for Random Forest = 0.83 ± 0.006, Linear = 0.89, for Kernel K-nearest with 2 neighbours Knn(2) = 0.75, for Kernel K-nearest with 3 neighbours Knn(3) = 0.79, and for Gaussian process approach = 0.83). We also included results from combining both datasets to a single one and obtained the following results in term of ROC AUC: linear SVM = 0.89 ± 0.001, SVM with radial basis function = 0.80 ± 0.001, Random Forest = 0.76 ± 0.004, Linear = 0.87, Kernel K-nearest with 2 neighbours Knn(2) = 0.74, Kernel K-nearest with 3 neighbours Knn(3) = 0.74, Gaussian process = 0.81.

**Table 2:** AUC results for machine-learning methods, averaged over 100 random seeds (1 standard error).

	SVM linear (mean ± SD)	SVM rbf (mean ± SD)	Random Forest (mean ± SD)	Linear	Knn (2n)	Knn (3n)	GP
Dataset1	0.71 ± 0.001	0.45 ± 0.025	0.70 ± 0.008	0.74	0.63	0.68	0.64
Dataset2	0.88 ± 0.001	0.80 ± 0.007	0.83 ± 0.006	0.89	0.75	0.79	0.83
Both datasets	0.89 ± 0.001	0.80 ± 0.001	0.76 ± 0.004	0.87	0.74	0.74	0.81

*SVM* Support Vector Machine, *SVM rbf* Support Vector Machine with radial basis function, *Kernels. Knn (2n)* K-nearest with 2 neighbours, *Knn (3n)* K-nearest with 3 neighbours, *GP* Gaussian Process.

### Illustrative health economic model

By averaging the classification performance of the SUVRc from linear and non-linear analyses (both datasets) we estimated it is capable of identifying nearly one half of the (33% of) patients destined to prove unresponsive to first-line treatment.

By combining this estimate with our model cost assumptions (see “Methods”), and assuming a specificity of 95% (i.e., 5% misclassification of responders), we obtained a total saving of £3400/patient. With these assumptions, the breakeven sensitivity of the classification test is 26% (Supplementary Fig. 5). While sensitivity modulates the potential savings of the biomarker, specificity has a strong effect on costs (Supplementary Fig. 6). By reducing the specificity of the test to 80%, the breakeven sensitivity will increase to 34.2%, and, at a sensitivity of 50%, there will be a saving per patient of ~£2000. Note that with both specificity and sensitivity at 50% (chance level), the biomarker would stop being economically favourable (Supplementary Table 8).

## Discussion

Our first main finding is that a dynamic [^18^F]FDOPA PET scan is able to identify 40–60% of treatment non-responsive patients with a specificity of 100%. Our second main finding is that a simplified 10 min [^18^F]FDOPA PET imaging scan acquisition shows good test-retest reliability (ICC = 0.76–0.91), good agreement with the dynamic [^18^F]FDOPA PET, and is able to identify 40–60% of treatment non-responsive patients with a specificity of 100%. Based on this, our economic modelling indicates the use of the simplified [^18^F]FDOPA PET imaging to guide early use of clozapine has a potential cost saving of £3.4 million per 1000 patients relative to current practice. However, our illustrative health economic analysis considered only direct health service costs; if social costs were included (e.g., criminal justice service costs), the potential cost savings is likely to be greater [72]. These findings extend previous molecular imaging studies reporting presynaptic striatal dopamine measures are associated with treatment response [20, 25, 27, 28, 32] by showing the clinical potential of these approaches if used in clinical practice.

In absolute terms the machine-learning analyses showed similar performance to the ROI analyses, although the linear and support vector machine classifiers were marginally superior to the ROI classifier for the medicated patient dataset (*Dataset2*). Recent meta-analyses of machine-learning methods have shown no or limited improvements over logistic regression in a medical context [73]. Our findings are generally consistent with these findings but extend them by showing that a logistic regression model shows no appreciable improvement in classification relative to the simple averaging approach. In our case the small size of the datasets compared with the high dimensionality of the features is likely to cause machine-learning and other data-driven methods to fail to capture reliable signal without further assumptions [74]. Further work to validate decision algorithms should be repeated considering larger samples following the guidelines of Steyerberg [75] and the PRoGRESS group [76].

The test-retest reproducibility and reliability of the simplified index of FDOPA uptake are in line with the ICC (0.68–0.94) and %VAR (0.7) values for *K*_i_^cer^ in the striatal subdivisions reported in Egerton et al. using a full dynamic acquisition [39]. These values indicate that the simplified imaging approach does not sacrifice reliability or increase variability compared to the standard, dynamic method.

[^18^F]FDOPA PET imaging is not the first method that has been proposed for early identification of treatment response in psychosis. Peripheral biomarkers (e.g., insulin levels, metabolite levels in blood and urine) have been shown to be linked to treatment response in psychosis [77, 78], but have not been consistent among studies [79]. Moreover, these findings were not validated using a cohort of responders and non-responders [78]. Mondelli et al. showed that lower cortical awakening response and increased levels of inflammatory markers are associated with poor treatment response in first-episode psychosis patients [80]. However, this study does not report any classification analysis and it has not been replicated to date. In the domain of neuroimaging, structural techniques have shown that differences in gyrification, cortical thickness and asymmetry are associated with subsequent response to antipsychotic treatments [81, 82]. Reduced white matter integrity has also been linked to non-response to treatment in patients with first-episode psychosis [83], and functional connectivity of the ventral tegmental area has been associated with treatment response [84]. Elevated anterior cingulate cortex glutamate levels have also been reported to be associated with treatment resistance compared to treatment-responsive schizophrenia patients [85]. However, none of these studies reported a validation analysis of the predictive power of the proposed measure, AUC values nor sensitivity/specificity classification. Moreover, these studies include one dataset only. Thus, our study extends these previous peripheral biomarker and neuroimaging studies by including these analyses and considering two independent datasets.

A recent study provided evidence that individual differences in striatal functional connectivity predict response to antipsychotic treatment in acutely psychotic patients (AUC: 0.78, sensitivity: 80% and specificity: 75%) [86]. Our potential biomarker showed predictive power that was higher than this (AUC of 0.89). However, as they did not report sensitivity at 100% specificity or cost analyses, it is not clear how it performs on these metrics. It would be useful to directly compare both approaches in a future study.

### Strengths and limitations

Strengths of our work include the use of an in vivo measure of the pathophysiology of psychosis relevant to the mechanism of action of antipsychotic treatments, and that we were able to test the [^18^F]FDOPA PET imaging approach in two independent datasets and test reliability of the simplified protocol in a third dataset. However, by the very nature of the cohorts recruited (first-episode psychosis, people with established illness) the criteria for treatment response were different in the two datasets, as outlined in “Methods”. This is reflected in the difference in sensitivity, 39% vs. 79%. It has also been shown that demographics and environmental factors, such as age, gender, smoking, ethnicity and childhood trauma, may influence dopamine synthesis capacity [87, 88]. The relative contribution of state and trait related factors to dopamine alterations in psychosis remains to be determined [89]. Nevertheless, the pathological mechanisms underlying the disorder should not be confused with the capacity of the biomarker to predict a clinical response to treatment. An ideal biomarker should return accurate results irrespective of the history of the patients to which it is applied [90]. Moreover, whilst our sample sizes are large for PET imaging studies (*N* = 84 individuals pooled together from 3 different studies), it is important to recognise that the samples are relatively modest, and our findings will require testing in other samples and settings to determine their generalisability, and if clinical-demographic factors influence results, before they can be translated into routine clinical practice. Assuming a ROC AUC = 0.7, a marginal error of 10%, a conservative frequency of non-responding patients of 20% and a drop-out rate of 10%, a power calculation in easyROC (v1.3.1) [91] indicates it would take 84 first-episode patients to test the biomarker accuracy to predict treatment response in a prospective study with power = 0.80, and *α* = 0.05.

Of particular interest might be the use of PET-MR scanners to obtain hybrid samples that could provide additional information to include in the presented machine-learning tool. This could be particularly relevant for those MRI modalities that provided complementary information on the dopamine system, like neuromelanin-MRI [92] or dopamine enriched functional connectivity [93, 94]. Finally, we calculated SUVRc retrospectively from a 10-min time frame of a dynamic PET scan and this warrants testing in a prospective study. The AUC in the receiver operating curve analyses were between 0.66 and 0.87 and statistically higher than chance performance.

Performance of this simplified FDOPA PET is dependent on the experimental design, including the co-administration of carbidopa and entacapone. These are recommended to enhance the specific brain FDOPA signal [27] by reducing metabolite production [95], but future studies would be useful to determine the importance of this for classification accuracy. All the datasets were collected by the same research group and used the same experimental design, imaging acquisition and analysis pipeline. Future studies should include multi-centre acquisitions to investigate the generalisability of the biomarker across settings.

In our preliminary economic analysis, we set specificity at 95% and found substantial cost savings. A prospective study is now needed to determine if the biomarker can achieve 95% specificity in routine clinical practice. Nevertheless, our further analyses show that the biomarker has the potential to be cost effective with specificity and sensitivity values as low as 0.6. This is in line with the findings of a recent modelling study of a test to identify patients for clozapine [59], which showed that a stratified test with 60% sensitivity and specificity is still cost effective compared to no test for people with first-episode psychosis who failed a first-line antipsychotic. Whilst we accounted for the cost of managing neutropenia, we did not factor in the costs of other side effects. However, in contrast with the risk of neutropenia, the comparative risks of other side effects of clozapine and first-line antipsychotics are relatively similar [65, 96, 97], suggesting that there is unlikely to be a large cost differential. Notwithstanding this, a limitation of our economic analysis is that the costs of side effects have only been partially considered.

### Implications of our findings

Our results have two main implications. The first is that in the two examined sampled we were able to identify 50% of non-responders without overlap with responders; these results need to be replicated in future studies. This could enable early use of clozapine, which is associated with improved outcomes [98]. Studies have reported that early intervention for psychosis via theragnostic biomarkers could lead also to long-term economic benefit [72, 99–101]. Hospitalisation costs are the main component of the higher total healthcare costs associated with treatment-resistant schizophrenia [102], with approximately half of these costs attributable to hospital admissions [8]. Health economic evidence suggests that the use of clozapine has the potential to improve the use of health and social service resources in treatment-resistant schizophrenia [103], through shorter inpatient admissions [11, 104], lower rates of relapse and reduced rehospitalization [105–108]. A cost-benefit model study within the Veterans Health Administration in the USA compared costs with clozapine use and non-use in treatment-resistant schizophrenia. It identified that, if 20% of treatment-resistant cases started clozapine, this would be associated with an average annual reduction in health costs of $22,444 per patient treated with clozapine [109].

Our preliminary economic analysis indicates that screening test with [^18^F]FDOPA PET would lead to an average healthcare saving of £3400 per patient with schizophrenia. However, these potential benefits need to be tested in a prospective study and assume that all non-responders identified go on to clozapine. Another consideration for translation is the practicalities of [^18^F]FDOPA PET scanning. Practicability of PET in first-episode psychosis was demonstrated both in research samples [34, 40–42], suggesting that 10-min [^18^F]FDOPA PET scan could be potentially well tolerated, but this needs evaluating in clinical settings. In support of [^18^F]FDOPA PET practicability in a clinical environment the key evidence is:

1. FDOPA is already approved by the European Medicines Agency as a diagnostic agent (EMA/729548/2018), meaning its manufacture is standardised, facilitating introduction to health services.
2. FDOPA imaging is already approved by the FDA for the diagnosis of Parkinson’s Disease (2019, ID: 4504654). As such, the extension to another indication would be relatively straightforward.
3. FDOPA has a sufficiently long half-life to enable it to be produced in a distribution centre, and then delivered to hospitals. This is well established for clinical oncology PET imaging with other tracers like FDG.

If this approach is to be implemented, parameter values and operating procedures would need to be established for a range of scanners and sites. The former could be done using normative data from healthy volunteers or brain phantoms scanned across different centres and scanners. The second implication is that [^18^F]FDOPA PET imaging could be used to identify non-responders for inclusion in trials of novel treatments for non-response. This could be used to enrich trials for patients who might respond to a novel therapy.

## Conclusions

Our study showed that a short [^18^F]FDOPA PET imaging protocol provides reliable and reproducible measures of dopamine synthesis and that it and the full dynamic [^18^F]FDOPA PET imaging protocol can be used to distinguish patients with schizophrenia who are unlikely to respond to first-line antipsychotic treatments from those who will respond at first episode. The classification accuracy of the proposed simplified imaging method is comparable to that from a dynamic [^18^F]FDOPA PET scan. These results support the development of the short [^18^F]FDOPA scan as a pathophysiologically relevant biomarker to guide therapy choice for patients with psychosis.

## Funding and disclosure

This study was funded by Medical Research Council-UK (no. MC-A656-5QD30), Maudsley Charity (no. 666), Brain and Behaviour Research Foundation, and Wellcome Trust (no. 094849/Z/10/Z) grants to OH and the National Institute for Health Research (NIHR) Biomedical Research Centre at South London and Maudsley NHS Foundation Trust and King’s College London. The views expressed are those of the author(s) and not necessarily those of the NHS, the NIHR or the Department of Health. MV is funded by the National Institute for Health Research Biomedical Research Centre at South London and Maudsley National Health Service Foundation Trust and King’s College London, by the Wellcome Trust Digital Award 215747/Z/19/Z. SJ is funded by the National Institute for Health Research Biomedical Research Centre at South London and Maudsley National Health Service Foundation Trust and King’s College London, and a JMAS SIM Fellowship from the Royal College of Physicians, Edinburgh. OH has received investigator-initiated research funding from and/or participated in advisory/speaker meetings organised by Angellini, Astra-Zeneca, Autifony, Biogen, Boehringer-Ingelheim, Eli Lilly, Heptares, Invicro, Jansenn, Lundbeck, Lyden-Delta, Mylan, Neurocrine, Otsuka, Sunovion, Rand, Recordati, and Roche. Neither OH nor his family have been employed by or have holdings/a financial stake in any pharmaceutical company. MV has received consulting honoraria from GSK. OH and MV hold a patent application for the use of dopamine imaging as a prognostic tool. SJ has received honoraria for educational talks from Sunovian and his employer (KCL) has received honoraria for educational talks he has given for Lundbeck. SJ is also Co-PI for a drug trial for a product manufactured by Alkermes. All the other authors have nothing to disclose.

## Supplementary information

Supplementary Material
