# Supplementary material for: A potential biomarker for treatment stratification in psychosis: evaluation of an [18F] FDOPA PET imaging approach
Source: Neuropsychopharmacology. 2020 Sep 22;46(6):1122–32. doi: 10.1038/s41386-020-00866-7 (PMC8115068; doi:10.1038/s41386-020-00866-7)
Supplement: Supplementary file 1 — Supplementary Material [file 41386_2020_866_MOESM1_ESM.docx]

**A POTENTIAL BIOMARKER FOR TREATMENT STRATIFICATION IN PSYCHOSIS: EVALUATION OF A NEW, SIMPLIFIED FDOPA PET IMAGING APPROACH**

**Supplementary Material**

# ECONOMIC MODELLING

See references [56 – 65] in the main manuscript for model assumptions and associated costs.

**REPRESENTATIVE POPULATION**

NP = 1000;

**BIOMARKER PERFORMANCE and COST OF IMPLEMENTATION**

Performance

Sensitivity = 0.50; (Fraction of Non-Responders correctly classify before treatment)

Specificity = 0.95; (Fraction of Responders correctly identify)

FP = 1-Specificity; (Fraction of Responders switch to Clozapine)

Cost of implementation

CostPerScan = 3000; (£ Cost per PET scan)

FailureRate = 0.10; (Fraction of failed PET scans over total PET scans which still required to be paid)

**CostBiomarkerImplementation = NP*CostPerScan*(1+FailureRate);**

**ECONOMIC SAVING OF BIOMARKER**

Assumptions

NR = 0.33; (Fraction of Non-Responders over total number of psychotic patients)

ClozapineEffectiveTreatment = 0.5; (Fraction of Non-Responders responding to Clozapine)

EconomicalConveniencePerYear = 24000; (£/year, saving per patient from active psychosis to remission)

TimeToClozapine = 1/12; (years, time to get Clozapine with the biomarker)

DelayToClozapine = 4; (years, time to get Clozapine without the biomarker)

Value of the Biomarker switching early to clozapine

**TotalBiomarkerValue =**

**NP * NR * Sensitivity * ClozapineEffectiveTreatment * EconomicalConveniencePerYear * (DelayToClozapine-TimeToClozapine);**

**COST OF CLOZAPINE TREATMENT**

For Non-Responders

DailyCostClozapine = 1.56; (£, daily cost of Clozapine)

AnnualCostClozapineDrug = DailyCostClozapine*365; (£/year, cost of Clozapine treatment)

AnnualCostClozapineMonitoringRegime = 37.57*12; (£/year, cost of Clozapine monitoring service, 1 visit/month)

CostClozapineSideEffect = 469.48; (£, cost of treating neutropenia)

ProbabilityClozapineSideEffect = 0.03; (Probability of developing neutropenia in a year of treatment)

AnnualCostClozapineSideEffect = CostClozapineSideEffect * ProbabilityClozapineSideEffect;

AnnualCostOfClozapineNonResponder = AnnualCostClozapineDrug + AnnualCostClozapineMonitoringRegime +

AnnualCostClozapineSideEffect;

**AdditionalCostBiomarker_ClozapineEarly2NonResponder =**

**NP * NR* Sensitivity * AnnualCostOfClozapineNonResponder * (DelayToClozapine-TimeToClozapine);**

For Responders

DailyCostNonClozapine = 0.85; (£, average price of standard antipsychotic, weighted by market share)

AnnualCostOfNonClozapine = DailyCostNonClozapine*365; (£/year, cost of standard antipsychotic treatment)

AnnualCostClozapineMonitoringSetUpRegime = 37.57*2*12; (£/year, cost of Clozapine monitoring service, 2 visit/month)

AnnualCostOfClozapineResponder = (AnnualCostClozapineDrug-AnnualCostOfNonClozapine) + AnnualCostClozapineMonitoringSetUpRegime + AnnualCostClozapineSideEffect;

TimeOfPrediction = 10; (years, model time of prediction)

**AdditionalCostBiomarker_ClozapineEarly2Responder =**

**NP * (1-NR) * FP * AnnualCostOfClozapineResponder * TimeOfPrediction;**

Total cost of Clozapine treatment

**TotalCostClozapineTreatment =**

**AdditionalCostBiomarker_ClozapineEarly2NonResponder + AdditionalCostBiomarker_ClozapineEarly2Responder;**

**SAVINGS**

**BUDGET = TotalBiomarkerValue - (CostBiomarkerImplementation+TotalCostClozapineTreatment);**

# SUPPLEMENTARY TABLES

**Table 1** Pearson’s correlation coefficients (r) between the reference K_i_^cer^(95 min) and K_i_^cer^(60min) in the whole striatum and its functional subdivisions.

|  | **Pearson correlations between reference K_i_^cer^ estimates (computed using 0-95 min dynamic PET) and K_i_^cer^ estimates using a short 0-60 min dynamic PET scan** |
| --- | --- |
| **Whole Striatum** | r= 0.86 |
| **Associative Striatum** | r= 0.83 |
| **Limbic Striatum** | r= 0.67 |
| **Sensorimotor Striatum** | r= 0.81 |

**Table 2** Intra-class correlation coefficient (ICC) for K_i_^cer^(60min) in the whole striatum and its functional subdivisions.

|  | **K_i_^cer^ estimates using 0-60 min dynamic PET scan**  (ICC single measures, ICC average measures, sig.) |
| --- | --- |
| **Whole Striatum** | 0.83, 0.91, **0.002*** |
| **Associative Striatum** | 0.78, 0.88, **0.006*** |
| **Limbic Striatum** | 0.22, 0.36, **0.281** |
| **Sensorimotor Striatum** | 0.92, 0.95, **0.0001*** |

**Table 3** Study datasets

|  | Dataset1* | | | | Dataset2** | | | Dataset3*** | |
| --- | --- | --- | --- | --- | --- | --- | --- | --- | --- |
|  | **Controls**  **(N=14)** | **Responders**  **(N=13)** | **Non-responders**  **(N=13)** | **Controls**  **(N=12)** | | **Responders**  **(N=12)** | **Non-responders**  **(N=12)** | **Controls**  **(N=8)** |  |
| Age (mean±SD) | 24.3±4.62 | 24.4±3.02 | 26.2±5.78 | 44.2±8.9 | | 44±11.9 | 45.7±9.8 | 23.6±3.5 |  |
| Gender  (M;F) | 10;4 | 10;3 | 12;1 | 5;7 | | 6;6 | 5;7 | 5;3 |  |

* Dataset1 includes only patients with first-episode psychosis, unmedicated/minimally treated with a history of disease < 2 years.

** Dataset2 includes only medicated patients with history of disease > 16 years.

*** Dataset3 is a test-retest dataset.

**Table 4** Spearman’s correlation analysis (rho) between the standard dynamic measure of dopamine synthesis capacity (Ki^cer^, 1/min) and the simplified index of FDOPA uptake (SUVRc, unitless) calculated at different time intervals. This analysis refers to whole striatum and functional striatal subdivisions and considers both patient and control groups of Dataset1 and Dataset2. Significant correlations remain statistically significant after log transformation of SUVRc estimates.

|  | Whole Striatum | | Sensorimotor | | Limbic | | Associative | |
| --- | --- | --- | --- | --- | --- | --- | --- | --- |
|  | **Dataset1** | **Dataset2** | **Dataset1** | **Dataset2** | **Dataset1** | **Dataset2** | **Dataset1** | **Dataset2** |
| 60 minutes | 0.86** | 0.83** | 0.89** | 0.79** | 0.80** | 0.87** | 0.85** | 0.84** |
| 75 minutes | 0.87** | 0.83** | 0.86** | 0.85** | 0.83** | 0.87** | 0.86** | 0.85** |
| 85 minutes | 0.82** | 0.83** | 0.85** | 0.84** | 0.75** | 0.80** | 0.84** | 0.84** |

** p<0.0001

**Table 5** Spearman’s correlation analysis (rho) between the standard dynamic measure of dopamine synthesis capacity (Ki^cer^, 1/min) and the simplified index of FDOPA uptake (SUVRc, unitless) in whole striatum and functional striatal subdivisions. This analysis considers only patients (responder and non-responders) of Dataset1 and Dataset2. Significant correlations remain statistically significant after log transformation of SUVRc estimates.

|  | Whole Striatum | | Sensorimotor | | Limbic | | Associative | |
| --- | --- | --- | --- | --- | --- | --- | --- | --- |
|  | **Dataset1** | **Dataset2** | **Dataset1** | **Dataset2** | **Dataset1** | **Dataset2** | **Dataset1** | **Dataset2** |
| 60 minutes | 0.79** | 0.79** | 0.87** | 0.74** | 0.77** | 0.84** | 0.78** | 0.85** |
| 75 minutes | 0.78** | 0.80** | 0.77** | 0.82** | 0.74** | 0.84** | 0.79** | 0.82** |
| 85 minutes | 0.67* | 0.80** | 0.71** | 0.82** | 0.60** | 0.83** | 0.76** | 0.81** |

** p<0.0001

* p<0.05

**Table 6** Cohen’s d effect size of the simplified index of FDOPA uptake (SUVRc, unitless) between responders and non-responders patients in striatum and functional striatal subdivisions for both Dataset1 and Dataset2.

|  | Whole Striatum | | Sensorimotor | | Limbic | | Associative | |
| --- | --- | --- | --- | --- | --- | --- | --- | --- |
|  | **Dataset1** | **Dataset2** | **Dataset1** | **Dataset2** | **Dataset1** | **Dataset2** | **Dataset1** | **Dataset2** |
| 60 minutes | 1.09 | 0.70 | 0.60 | 0.57 | 0.93 | 0.67 | 1.24 | 0.70 |
| 75 minutes | 0.98 | 0.79 | 0.46 | 0.76 | 0.66 | 0.66 | 1.32 | 0.79 |
| 90 minutes | 0.62 | 0.64 | 0.17 | 0.56 | 0.21 | 0.67 | 0.88 | 0.64 |

**Table 7** Percentage variability (%VAR, mean) and intra-class correlation coefficient (ICC) of the simplified index of FDOPA uptake (SUVRc) in striatum and functional subdivisions.

|  | Whole Striatum | | Sensorimotor | | Limbic | | Associative | |
| --- | --- | --- | --- | --- | --- | --- | --- | --- |
|  | **ICC** | **%VAR** | **ICC** | **%VAR** | **ICC** | **%VAR** | **ICC** | **%VAR** |
| 60 minutes | 0.79 | 3.30 | 0.82 | 3.60 | 0.76 | 2.70 | 0.77 | 3.40 |
| 75 minutes | 0.88 | 2.80 | 0.91 | 3.40 | 0.86 | 0.20 | 0.87 | 3.30 |
| 90 minutes | 0.8 | 2.60 | 0.76 | 3.30 | 0.82 | 1.40 | 0.8 | 3.10 |

**Table 8** Cost/Saving per patient (in £) assuming different levels of specificity and sensitivity. Green values are economically favourable, while red values are economically unfavourable

|  | | Specificity | | | | | |
| --- | --- | --- | --- | --- | --- | --- | --- |
|  |  | **0.5** | **0.6** | **0.7** | **0.8** | **0.9** | **1.0** |
| Sensitivity | **0.5** | -149.4 | 637.8 | 1,425.0 | 2,212.2 | 2,999.4 | 3,786.6 |
|  | **0.6** | 1,267.9 | 2,055.1 | 2,842.3 | 3,629.5 | 4,416.7 | 5,203.9 |
|  | **0.7** | 2,685.2 | 3,472.4 | 4,259.6 | 5,046.8 | 5,834.0 | 6,621.2 |
|  | **0.8** | 4,102.5 | 4,889.7 | 5,676.9 | 6,464.1 | 7,251.3 | 8,038.5 |
|  | **0.9** | 5,519.9 | 6,307.1 | 7,094.2 | 7,881.4 | 8,668.6 | 9,455.8 |
|  | **1.0** | 6,937.2 | 7,724.4 | 8,511.6 | 9,298.8 | 10,085.9 | 10,873.1 |

# SUPPLEMENTARY FIGURES

**Figure 1** Correlation analysis between the standard dynamic measure of dopamine synthesis capacity (Ki^cer^, min^-1^) and the simplified index of FDOPA uptake (SUVRc, unitless) in whole striatum at different time points after tracer injection. This analysis considers both patient and control groups of Dataset1 (**A**) and Dataset2 (**B**).

**Figure 2** Sensitivity analysis between the simplified index of FDOPA uptake (SUVRc) in whole striatum and the tracer-specific activity (SA [GBq/μmol]) and injected dose [MBq]. There was no correlation between SUVRc and both SA (p=0.97) and injected dose (p=0.89).

**Figure 3** Striatal (left column) and extra striatal (right column) FDOPA PET tissue contrast by using 5-minute (A and D), 10-minute (B and E) and 15-minute (C and F) acquisition window. Colour bar refers to FDOPA tracer activity. Extrastriatal maps are obtained by removing high uptake striatal voxels.

**Figure 4** Receiver Operating Characteristic (ROC) curves for the classification of patients into responders and non-responder groups using [18F]FDOPA PET imaging analysed using the simplified index of FDOPA uptake (SUVRc) or the standard dynamic measure of dopamine synthesis capacity (Ki^cer^) in the whole striatum and associative striatal subdivision. This analysis shows the results from Dataset1 (**A**) and Dataset2 (**B**).

***Figure 5 Economic modelling and sensitivity analysis*** *Cost per patient includes biomarker implementation, and additional costs to get Clozapine (treatment, monitoring and side effect) for all the patients tested positive with the biomarker. Saving per patient models the economic value of treating a non-responder patient with clozapine earlier than current clinical practice. Red area: economically unfavourable (Cost>Saving). Green area: economically favourable (Saving>Cost). Blue line: economic value at predicted biomarker performance (sensitivity=0.50, specificity=0.95)*

***
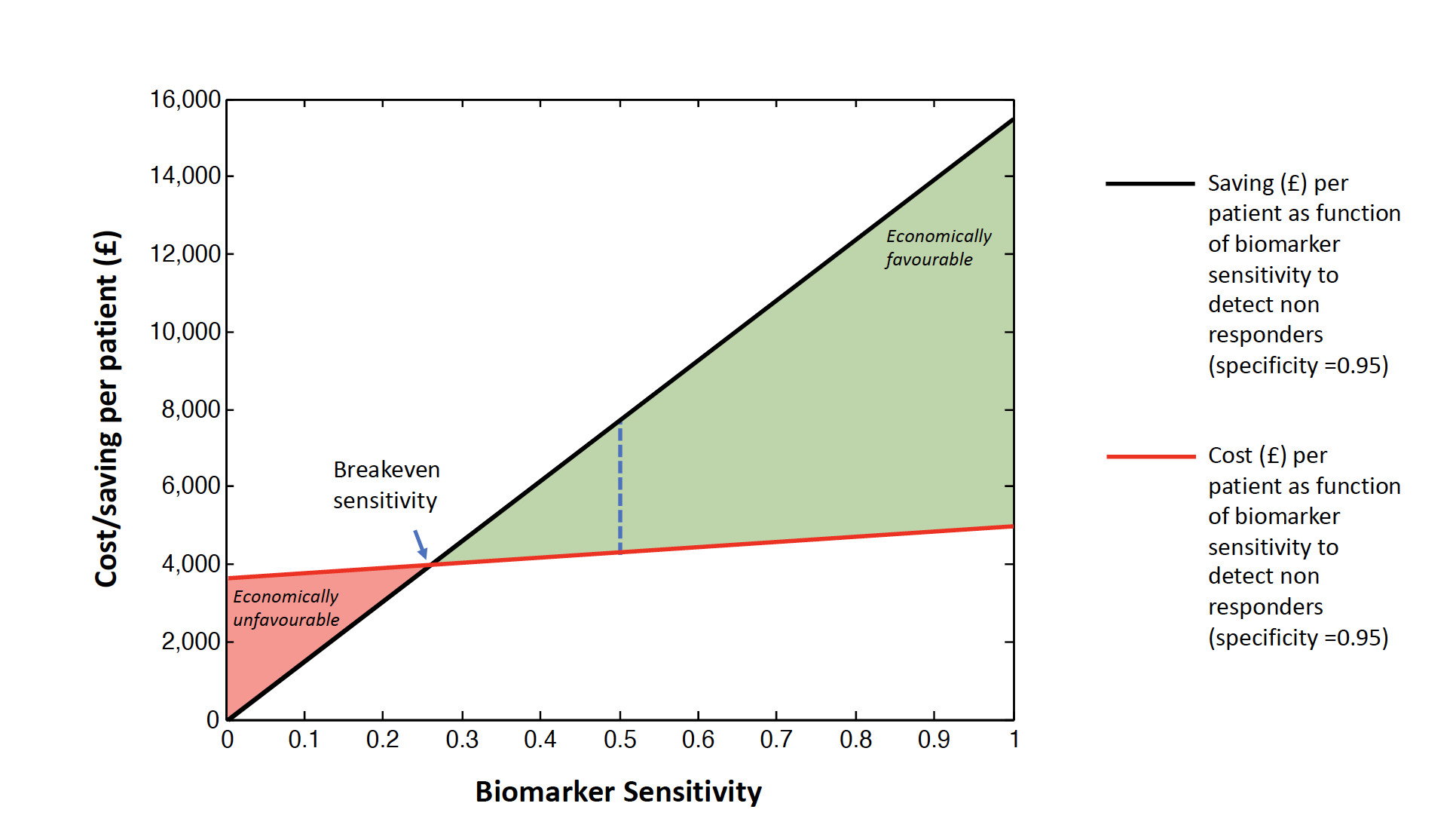
***

***Figure 6 Economic modelling and specificity analysis*** *Cost per patient includes biomarker implementation, and additional costs to get Clozapine (treatment, monitoring and side effect) for all the patients tested positive with the biomarker. Saving per patient models the economic value of treating a non-responder patient with clozapine earlier than current clinical practice. Red area: economically unfavourable (Cost>Saving). Green area: economically favourable (Saving>Cost). Blue line: economic value at predicted biomarker performance (sensitivity=0.50, specificity=0.95).*

**
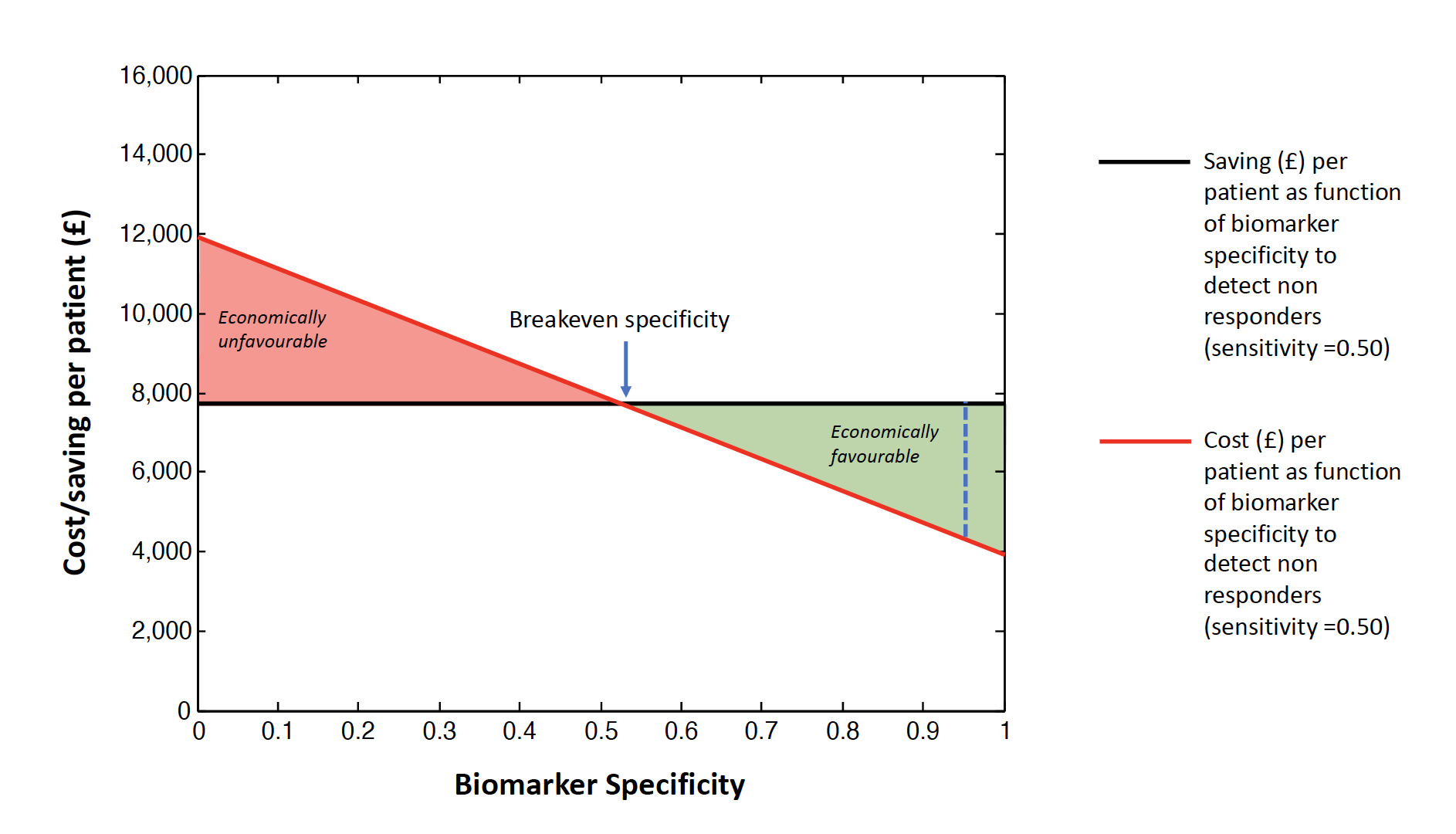
**
